# Malaria, Schistosomiasis and Soil Transmitted Helminth Burden and Their Correlation with Anemia in Children Attending Primary Schools in Kinshasa, Democratic Republic of Congo

**DOI:** 10.1371/journal.pone.0110789

**Published:** 2014-11-05

**Authors:** Junior R. Matangila, Joachim Yorokpa Doua, Sylvie Linsuke, Joule Madinga, Raquel Inocêncio da Luz, Jean-Pierre Van Geertruyden, Pascal Lutumba

**Affiliations:** 1 Département de Médecine Tropicale, Université de Kinshasa, Kinshasa, République Démocratique du Congo; 2 Department of Epidemiology, University of Antwerp, Antwerp, Belgium; 3 Institut National de Recherche Biomédicale, Kinshasa, République Démocratique du Congo; 4 Research Institute of Health and Society (IRSS), Université Catholique de Louvain, Brussels, Belgium; CEA, France

## Abstract

**Background:**

Anaemia reduces cognitive potential in school children, retards their growth and predisposes them to other diseases. As there is a paucity of data on the current burden of *P. falciparum*, *S. mansoni* and soil transmitted helminths (STH) infections and their correlation with schoolchildren’s anemia in the Democratic Republic of Congo (DRC), we collect these data.

**Methods:**

This study reports baseline data collected from a randomized controlled trial investigating the impact of IPT with SP and SP-PQ on anemia and malaria morbidity in Congolese schoolchildren (Trial registration: NCT01722539; PACTR201211000449323). *S. mansoni* and STH infections were assessed using kato-katz technique. Malaria infection and hemoglobin concentration were assessed using Blood smear and Hemocontrol device, respectively.

**Results:**

A total of 616 primary schoolchildren from 4 to 13 years old were enrolled in the study. The prevalence of *Plasmodium spp.* infection was 18.5% (95%CI:15.6–21.9). Amongst those infected, 24 (21%), 40 (35.1%), 40 (35.1%), 10 (8.8%), had light, moderate, heavy, very high malaria parasite density, respectively. Above 9 years of age (p = 0.02), male and history of fever (p = 0.04) were both associated with malaria infection. The overall prevalence of *S. mansoni* infection was 6.4% (95%CI:4.4–9.1). Girls were associated with *S. mansoni* infection (p = 0.04). *T. trichiura* was the most prevalent STH infection (26.3%), followed by *A. lumbricoides* (20.1%). Co-infection with malaria-*S. mansoni* and malaria-STH was, respectively, 1.5% (CI_95%_:0.7–3.3) and 6.4% (CI_95%_ 4.4–9.1). The prevalence of anemia was found to be 41.6% (95%CI:37.7–45.6) and anemia was strongly related with *Plasmodium ssp* infection (aOR:4.1; CI95%:2.6–6.5;p<0.001) and *S. mansoni* infection (aOR:3.3;CI95%:1.4–7.8;p<0.01).

**Conclusion:**

Malaria and *S. mansoni* infection were strongly associated with high prevalence of anemia in schoolchildren. Therefore, specific school-based interventions, such as intermittent preventive treatment or prophylaxis, LLITN distribution, anthelminthic mass treatment and micronutrient supplementation are needed to improve school children’s health.

## Introduction

Malaria, Schistosomiasis and Soil Transmitted Helminth (STH) infections compose a considerable disease burden in schoolchildren in developing countries. These parasitic infections show a similar geographic distribution and polyparasitism of *P.falciparum*, schistosomiasis and STH infections have been reported from various epidemiological settings in Africa [1]–[5].

In endemic malaria areas, the prolonged carriage of *P.falciparum* triggers the development of acquired immunity that controls blood-stage parasitaemia, thereby reducing clinical symptoms and life-threatening complications in older children and adults [6]. Asymptomatic *Plasmodium* infections, if untreated, persist and maintain malaria-induced inflammation that is commonly associated with iron deficiency anaemia (IDA) due to impaired intestinal iron absorption, impaired iron release from hepatocytes, and impairment of the recycling of iron derived from phagocytosis of parasitized red blood cells [7]. It has also been suggested that high levels of tumor necrosis factor (TNF) induces marked dyserythropoietic changes in the red cell precursors and increased erythrophagocytosis [8]–[10]. In Low Income Countries (LICs), more than half of the school-aged population suffers from anaemia and in Sub Saharan Africa, approximately 85 million school-aged children are affected. Anaemia reduces their cognitive potential, retards their growth, and predisposes them to other diseases [11]. Moreover, malaria accounts for about for 13–50% of all annual school absenteeism and consequently impairs educational achievements of children [12]. Schistosomiasis and STH also inflict significant adverse effects on health such as anemia, stunting, protein-calorie malnutrition, fatigue, and poor cognitive development. During Schistosomiasis, anemia results from four processes: iron deficiency due to extra-corporal blood loss; splenic sequestration; autoimmune hemolysis and inflammatory anemia [13].

Findings on the interaction of malaria and helminth infections on the health of children are controversial. Some studies have shown a protective effect of helminths on symptomatic malaria. Severe worm burden seems to suppress malaria symptoms [14], [15]. On the other hand, others have highlighted an increased severity and incidence of malaria during helminths co-infection [16], [17]. In addition an additive effect of *P. falciparum*, Schistosomiasis and/or STH infection on childhood anemia has been reported [18], [19].

The Democratic Republic of Congo (DRC) is at present one of the most affected countries by malaria [20]. Although high, the exact burden of malaria as well as other parasitic diseases is unclear partly because of lack of reliable surveillance system. Moreover, only 54.1% of the patients presenting fever and seeking health care were truly infected with malaria [21]. In fact, the DRC and Nigeria contribute to about 24% of non-malarial fever reported worldwide [21]. This highlights the urgent need of conducting studies to provide accurate data on the current burden of these diseases in DRC and others settings.

While a hand full of studies are dedicated to the groups most vulnerable for malaria such as children under the age of five and pregnant women, very little is known about the ongoing burden of asymptomatic *P. falciparum,* Schistosomiasis and STH infections, or their relationship with anemia in primary schoolchildren. The aim of this study was therefore to determine the prevalence of asymptomatic malaria, Schistosomiasis and STH infections in apparently healthy children attending schools in a semirural area in Kinshasa, DRC, as well as their relationship with anemia.

## Materials and Methods

### Study area

Kinshasa is one of the biggest cities in Africa and divided into 25 administrative zones or municipalities that cover about 9,965 km2. According to the national health policy, 25 Health Zones (HZs) roughly cover the 25 administrative zones. These HZs are also divided into Health Areas (HAs). Kimbanseke is the most densely populated peripheral municipality of Kinshasa housing 946, 372 inhabitants in 2004 [22]. The study was conducted in the Mokali HA. It is the most populated HA of Kimbanseke HZ, with an estimated 27,455 inhabitants. The participants were recruited at EP Boyambi and EP Likabo, two primary schools nearest to the regional health centre. Both schools were built by the Catholic Church and each school has 14 teachers and 12 classes. The esxpected number of schoolchildren was 650 per school at the beginning of the year.

### Study design

This study analyses cross-sectional, baseline data from an open label, randomised, controlled trial enrolling asymptomatic school children and investigating the impact of intermittent preventive treatment (IPT) with Sulfadoxine-Pyrimethamine (SP) and SP plus Piperaquine (PQ) on anemia and malaria morbidity in Congolese schoolchildren (Trial registration: NCT01722539; PACTR201211000449323 ). The trial design and protocol are described in detail elsewhere [23].

Briefly, asymptomatic primary school children were recruited from the 10^th^ to the 24^th^ November 2012. Children whose parents did not provide written informed consent or presenting fever, muscle aches or other symptoms suggestive for malaria at the time of enrolment were excluded. Children participating in another clinical trial were also excluded from the study. A structured questionnaire was used to obtain information on age, river contact, history of fever, diarrhea and abdominal pain.

### Laboratory analysis

#### Malaria diagnostic

Finger-prick blood specimens were taken for analysis of *Plasmodium* infection using microscopic examination for malaria parasites. Giemsa-stained thick blood smears (TBS) were used for microscopy. Blood slides were examined using light microscopy at 1000 × magnification. Hundred microscopic fields were examined in the thick smear before concluding that a blood slide was negative. All slides were read twice by experienced microscopists. If the discrepancy was greater than 15%, a third reader was used to confirm diagnosis. The parasite density per microliter of blood was calculated using the following formula: (Number of trophozoites x 8000)/Number of leucocytes. Parasite counts were utilized to classify the intensity of *Plasmodium spp.* infection into light, moderate, heavy, or very heavy infections respectively as followed: 1–499 parasites/µL, 500–1,999 parasites/µL, 2,000–9,999 parasites/µL and ≥10,000 parasites/µL.

#### Stool collection and parasite determination

Fecal samples were collected and taken to the laboratory of Parasitology of the University of Kinshasa for analysis using the Kato-Katz technique [24]. Slides were examined microscopically in a systematic manner 24 hours after preparation; *S. mansoni* and/or helminth eggs were counted and the number obtained was multiplied by the factor 24 in order to get the number of eggs per gram of feces (epg). Egg counts were utilized to classify infection intensities into light, moderate, or heavy infections respectively as followed: for *S. mansoni* 1–99 epg, 100–399 epg, ≥400 epg; *A. lumbricoides*, 1–4,999 epg, 5,000–49,999 epg and ≥50,000 epg; and for *T. trichiura*, 1–999 epg, 1,000–9,999 epg and ≥10,000 epg [25].

#### Urine analysis

Urine samples were analysed using the centrifugation method as described by Okanla [26]. Briefly, the samples were left to stand on the bench for about 30 min. Thereafter, the urine in each sample was drawn off leaving the last 10 mL in the bottle. The content of each bottle was shaken to suspend the sediment and was transferred into a 20 mL centrifuge tube. The tubes were centrifuged at 1 000 r/min for 5 min. The supernatant was discarded and the residue was put on a clean glass slide and examined under 10× objective lens of the microscope. The intensity of infection was estimated according to the number of eggs per 10 mL urine.

#### Measuring hemoglobin concentration

Hemoglobin (Hb) levels were determined using a HemoControl device (EKF Diagnostics, Germany). Anemia was defined by a hemoglobin concentration <11, <11.5 and <12 g/dL, respectively for schoolchildren of less than 5 years, 5–11.9 years and 12–14.9 years old. Anemia was classified as severe anaemia Hb <7 g/dl, moderate anaemia Hb: 7–9.9 g/dl and mild anaemia Hb: 10–11.4 g/dl. [27].

### Measuring malnutrition

Weight and height were measured in each child. With these measurements we calculate the following indicators using the WHO Anthroplus software: a) height-for-age Z-score (HAZ) to assess stunting in children 6 to 240 months old; b) weight-for-age Z-score (WAZ) to assess underweight in children of 6 to 120 months of age; and c) BMI-for-age Z-score (BAZ) to assess thinness in children of 6 to 60 months of age [28].

### Statistical analysis

The sample size was estimated on the basis of the expected additional impact on anemia of the intervention arm versus the comparator [23]. Data were entered and stored in Epi info7. Frequencies were used to assess the prevalence of asymptomatic malaria and anaemia in school children. Differences in mean values for continuous variables (e.g., HAZ, WAZ, BAZ, Hb, parasite densities) were assessed using the student t-test analysis. One-way ANOVA was used to analyze differences in Hb concentration mean of the study population by intestinal infection status and by infection intensity (negative, light and moderate-to-heavy) of each parasite species. Odds ratios (ORs), 95% confidence intervals (CIs) were calculated and p<0.05 values were considered to be statistically significant. Multivariate logistic regression models were constructed to identify factors associated with anemia in schoolchildren. Statistical analyses were done using SPSS statistical program, version 22 (SPSS, Chicago, IL, USA).

### Ethical considerations

The investigators agreed to conduct the present study in full agreement with the principles of the Declaration of Helsinki’ and subsequent relevant amendments. The study was approved by the Ethical Committees of the University of Antwerp, Belgium and of the School of Public Health, University of Kinshasa, DRCongo. Approval was also obtained from the Ministry of Health of DRCongo. Prior to the start of the project, special permission was obtained from the DRCongo Ministry of Education and the local health and education authorities of Biyela. A series of meetings were also held in the participating schools to explain the nature and purpose of the trial. Written informed consent (IC) was obtained from each parents or legal guardians of all children prior enrolment. An oral assent was obtained, as well, from children who were 12-years-old or older. All participants were administered albendazole (400 mg single oral dose) and praziquantel (40 mg/kg) against helminth infections. Children suffering from clinical malaria were treated according to national policy.

## Results

### Characteristics of schoolchildren

In total, 989 children aged between 4 and 13 years (median: 8 years and IQR:7.5–9.5 years) were assessed for eligibility in November 2012 of which 616 (62.3%) met the inclusion criteria and whose parents consented. A high frequency of refusal was found 324 (32.8%), with no real differences between schools in terms of percentage of parents refusing (Table 1). Children whose parents did not consent were similar to those included in regard to age, sex and class (data not shown). Forty nine, 49 children (4.9%) were excluded at baseline for the following raisons including: fever, history of multiple transfusions and weight <14 Kgs. Included children in the two primary schools were broadly similar in regard to age, sex, anthropometric indices, bed net use, contact with river, and symptoms histories characteristics. Anemia and parasitic infections were also similar between schools (Table 1). The mean height was 121 cm (SD:11.7) and the mean weight was 24.5 kg (SD 5.6). The most commonly reported symptoms were history of fever (31.0%;CI_95%_:27.4–34.9), abdominal pain (26.8%;CI_95%_ 23.4–30.5), diarrhea (15.3% CI_95%_ 12.6– 18.4), and bloody feces (11.2% CI_95%_ 8.9_–_14.0). Eleven percent of the children reported a history of blood transfusion (11.5%;CI_95%_ 9.2–14.4). Two hundred seventy seven school children (45%; IQR:41–49) were using the river as source of water to bathe or to wash their clothes.

**Table 1:** General characteristics of children attending two primary schools in Mokali heath area, in Kinshasa, 2012.

Variables	Boyambi School N = 558		% (95% CI)	Likabo School N = 431	% (95% CI)	P value	All N = 989	% 95% CI
Number of children (from class 1–5, %)	558		56.4 (53.3–59.5)	431	43.6 (40.5–46.7)		989	–
Refused/non response (%)	168		30.1 (26.4–34.1)	156	36.2 (31.5–40.8)		324	32.8 (28.3–36.7)
Excluded children (%)	29		5.2 (3.6–7.5)	20	4.6 (2.9–7.2)		49	4.95 (3.7–6.6)
Excluded for fever (%)	20		3.6 (2.3–5.6)	14	3.3 (1.9–5.5)		34	3.4 (2.4–4.8)
Excluded for multiple transfusion (%)	6		1.1 (0.44–2.5)	4	0.93 (0.30–2.5)		10	1.0 (0.51–1.9)
Excluded for Weight <14 Kgs (%)	3		0.54 (0.14–1.7)	2	0.46 (0.08–1.9)		5	0.51 (0.19–1.3)
Number of included children (%)	360		58.4 (54.4–62.4)	256	41.6 (37.7–45.6)		616	62.3 (57.3–67.7)
Female(%)		170	47.2 (42–52.5)	128	50 (43.7–56.3)		298	48.4 (44.4–52.4)
Median Age (IQR, years)	8 (7–9)		–	7 (7–8)	–	0.97	8 (7.5–9.5)	–
Mean weight (SD, Kgs)	24.56 (5.9)		–	24.51 (5.2)	–	0.91	24.53 (5.6)	–
Mean Height(SD, Cm)	125.9 (12.7)		–	126.5 (10.1)	–	0.53	126.2 (11.7)	–
Mean Hb (SD, g/dl)	11.54 (1.2)		–	11.64 (1.3)	–	0.32	11.6 (1.2)	–
*Anthropometric indicators of malnutrition*								
WHZ <−2 Z-score	5		1.4 (0.51–3.4)	1	0,39 (0.01 −2.2)		6	0.97 (0.4 −2.2)
WAZ <−2 Z-score	5		1.4(0.51 −3.4)	1	0.39 (0.01 −2.2)		6	0.97 (0.4 −2.2)
HAZ <−2 Z-score	1%)		0.28 (0.01 −1.8)	0	-		1	0.16 (0.01–1.1)
Anaemia (%)	157		43.6 (38.5 −48.9)	100	39.1 (33.1–45.3)		257	41.7 (37.8–45.7)
Severe anaemia (Hb <7 g/dl; %)	0		-	1	1 (0.03–5.5)		1	0.4 (0.01–2.2)
Moderate anaemia (Hb: 7–9.99 g/dl;%)	38		24.2 (17.7–31.7)	24	24 (16 −33.6)		62	24.1 (19–29.8)
Mild anaemia (Hb:10–11.49 g/dl; %)	119		75.8 (68.3–82.3)	75	75 (65.3–3.1)		194	75.5 (69.8–80.6)
*Symptom histories*								
History of fever(%)	100		27.8 (23.3–32.8)	91	35.5 (29.7–41.8)		191	31 (27.4–34.9)
History of diarrhea (%)	59		16.4 (12.8–20.7)	35	13.7 (9.7 −18.5)		94	15.3 (12.6 −18.4)
History of abdominal pain (%)	92		25.6 (21.2–30.5)	73	28.5 (23.1–34.5)		165	26.8 (23.4–30.5)
History of bloody feces	33		9.2 (6.5 −12.8)	36	14.1 (10.1–18.9)		69	11.2 (8.9–14)
History of transfusion	47		13.1 (9.8 −17.1)	24	9.4 (6.1–13.6)		71	11.5 (9.2–14.4)
History of cough (%)	70		19.4 (15.6 −24)	72	28.1 (22.7–34.1)		142	23.1 (19.8 −26.6)
History of itch (%)	51		14.2 (10.8–18.3)	52	20.3 (15.6–25.8)		103	16.7 (13.9–20)
*Parasitic infections*								
Malaria infection (%)	77		21.4 (17.3 −26.1)	37	14.5 (10.4–19.4)		119	18.5 (15.6 −21.9)
*Schistosoma mansoni* infection (%) N = 457	21		7.2 (4.5–10.8)	8	4.9 (2.1–9.4)		29	6.4 (4.4–9.1)
STH infection (%) N = 457	96		32.8 (27.4 −38.5)	54	32.9 (25.8–40.7)		150	32.8 (28.6 −37.4)
*Other characteristics*								
Contact with river (%)	175		48.6 (43.4 −53.9)	102	39.8 (33.8–46.1)		277	45 (41–49)
Bednet ownership (%)	60		17.1 (13.4–21.6)	58	24.2 (19–30.2)		118	20.3 (16.9 −23.6)
Bednet use (%)	37		61.7 (48.2 −73.9)	44	75.9 (62.8 −86.1)		81	68.6 (59.5–76.9)

EP: Ecole primaire; 95% CI: 95% confidence interval; Hb : haemoglobin.

### Excluded symptomatic children

A total of 34 (3.4%;CI_95%_: 2.4–4.8) children presented fever with a mean temperature of 38.6 (SD 1.2). Blood smear, hemoglobin concentration and stool examination were performed, before they were treated according to the national policy. The prevalence of malaria parasitaemia amongst febrile children was 41.2% (CI_95%_:24.7–59.3). The median malaria parasite density was 9840/µL (CI_95%_:4660–14480). No *S. mansoni* infection was diagnosed in symptomatic children (Table 2). However, the frequencies of STH infections were similar in both symptomatic and asymptomatic children (Table 3). The prevalence of anemia in this group was found to be 47.1% (CI_95%_ :29.8–64.9) (Table 2).

**Table 2:** General characteristics of symptomatic children, excluded from the trial.

Characteristic	N 34	%	(95%CI)
Mean Temperature (SD, °C)	38.6 (1.2)		
Median age (IQR, years)	8 (7–9)-	–	–
Mean height (SD, cm)	134 (10.1)	–	–
Mean weight (SD, kgs)	26.0 (4.84)	–	–
Mean Hb (SD, g/dl)	11.44 (0.99)	–	–
*Symptoms*			
Fever	34	100%	–
History of diarrhea	3	8.8%	(1.9–23.7)
History of abdominal pain	8	23.5%	10.8–41.2
History of bloody feces	3	8.8%	1.9–23.7
History of transfusion	3	8.8%	1.9–23.7
History of cough	7	20.6%	8.7–37.9
History of itch	7	20.6%	8.7–37.9
*Other characteristics*			
Contact with river	11	32.4%	17.4–50.5
Ownership of bed net	4	18.2%	5.2–40.3
Use of bed net	4	18.2%	5.2–40.3
*Anthropometric indicators of malnutrition*			
WHZ <−2 Z-score	1	2.9%	0.07–15.3
WAZ <−2 Z-score	1	2.9%	0.07–15.3
HAZ <−2 Z-score	0	–	–
Anaemia (%)	**16**	**47.1%**	**29.8–64.9**
Severe anaemia (Hb <7 g/dl; %)	–	–	–
Moderate anaemia (Hb: 7–9.99 g/dl;%)	1	6.3%	0.16–30.2
Mild anaemia (Hb:10–11.49 g/dl; %)	15	93.8%	69.8–99.8

**Table 3:** Frequencies of parasitic infections, in children attending two primary schools in Mokali heath semi-rural area, in Kinshasa, 2012.

	Asymptomatic children (N = 616)				Symptomatic children (N = 34)				
	N	%	CI95%	PD; Median, IQR	N	%	CI95%	PD; Median, IQR	P value
Parasitic infections									
***Plasmodium spp*** **. infection (TBS) (N = 616^a^/34^b^)**	**114**	**18.5**	**15.6–21.9**	**1478 (557–5279)**	**14**	**41.2**	**24.7–59.3**	**9840 (4660–14480)**	**0.01**
Light parasitaemia 1–499 parasites/µL	24	21	14–29.7		3	21.4	4.7–50.8		
Moderate parasitaemia 500–1,999 n/µL	40	35.1	26.4–44.6		1	7.1	0.2–33.9		
Heavy parasitaemia 2,000–9,999 n/µL	40	35.1	26.4–44.6		3	21.4	4.7–50.8		
Very heavy parasitaemia ≥10,000 n/µL	10	8.8	4.3−15.5		7	50.0	23–77		
***S. mansoni*** ** infection (Kato-Katz) (N = 457^a^/30^b^)**	**29**	**6.4**	**4.4–9.1**	**48 (24–76)**	**0**	–	–	–	–
Light parasite load 1–99	24	82.8	64.2–94.2		–				
Moderate parasite load 100–399	3	10.3	2.2–27.4		–				
High parasite load ≥400	2	6.9	0.9–22.8		–				
**Soil Transmitted Helminth infections**	**150**	**32.8**	**28.6–37.4**		**7**	**23.3**	**12.1–38.4**		
*** A. lumbricoides*** ** infection (Kato-Katz)**	**92**	**20.1**	**16.6−24.2**	**708 (300–2448)**	**3**	**10.0**	**2.1–26.5**	**1448 (48–2640)**	**0.1**
Light parasite load 1–4999 epg	82	89.1	80.9–94.7		3	100	–		
Moderate/heavy parasite load ≥5000 epg	10	10.9	5.3–19.1		0	_			
*** T. trichiura*** ** infection (Kato-Katz)**	**120**	**26.2**	**22.3–30.6**	**72 (58–2424)**	**4**	**13.3**	**3.8–30.7**	**96 (72–96)**	**0.2**
Light parasite load 1–999 epg	117	97.5	92.9–99.5		4	100	-		
Moderate/heavy parasite load ≥1000 epg	3	2.5	0.5−7.1		0				
***E. vermicularis*** ** (Kato-Katz)**	**1**	**0.2**	**0.01–1.4**	**24 (24–24)**	**0**				
**Co-infections (malaria - ** ***S. mansoni*** **)**	**7**	**1.5**	**0.7–3.3**		0				
**Co-infections (malaria - STH)**	**29**	**6.4**	**4.4–9.1**		2	6.7	0.72–19.7		
**Co-infection of at least two parasite species**	**79**	**17.3**	**15.3–20.8**		2	6.7	0.72–19.7		

PD: Parasite Density; ^a^: N° of asymptomatic children; ^b^: N° of symptomatic children, TBS: Tick Blood Smear.

### Asymptomatic malaria infection

**Figure 1:**
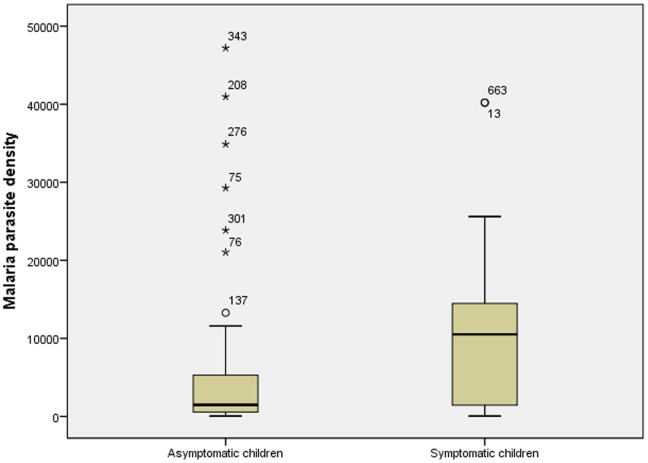
Difference of parasite density median between symptomatic and asymptomatic children.

The prevalence rate of *Plasmodium spp.* infection was 18.5% (CI_95%_:15.6–21.9) and was found to be significantly lower than in symptomatic children. *P. falciparum* was the most prevalent species (98.2%;CI_95%_:93.8–99.8). *P.malariae* was diagnosed in two children. In those with parasitemia, the median parasite density was 1478/µl (IQR:557–5279) and was also significantly lower than 9840 parasites/µL found in symptomatic children (p = 0.01) (Table 3.) (Fig. 1). The intensity of infection was found to be 24 (21%) with light, 40 (35.1%) moderate, 40 (35.1%) heavy, and 10 (8.8%) presenting very heavy parasite density (Table 1). Schoolchildren younger than 9 years old (aOR:0.47 CI_95%_ 0.26– 0.86; p = 0.02), and girls (aOR:0.57;CI_95%_:0.37–0.86; p<0.01) were less likely to have malaria infection. History of fever was an independent risk factor for malaria infection (aOR:1.6 CI_95%_ 1.0_–_2.4;p = 0.03) (Table 4). No significant difference in parasite density could be found between the children with or without history of fever (p = 0.09).

**Table 4:** Predictors for asymptomatic *P. falciparum infection*, *Schistosoma mansoni* infection and Soil Transmitted Helminth infections in children attending two primary schools in Mokali heath semi-rural area, in Kinshasa, 2012.

*Plasmodium spp*. Infection					*S. mansoni* infection			STH infections		
Characteristic		OR^a^ (95% CI)		P value	OR^b^ (95% CI )		P value	OR^c^ (95% CI )		P value
Age	4–9	0.47 (0.26–0.86)		0.01*	–	–	–	0.52	0.29–0.97	0.04*
	10–13	1			–			1		
Sex	Female	0.57	0.37–0.86	<0.01*	2.4	1.1–5.4	0.04*	0.62	0.41–0.92	0.02*
	Male	1			1			1		
History of fever	Yes	1.6	1.0–2.4	0.04*	–	–	–	NA		
	No	1			–					
Contact with river	Mango river	NA			2.7	1.2–6.0	0.02*	NA		
	Other river				1.0	0.5–2.1	0.9			
	No				1					

NA - Not applicable, OR- odds ratio, CI- Confidence Interval.

a Adjusted for: bed net use, *S. mansoni* and STH infections.

b Adjusted for: age, history of abdominal pain and diarrhea.

c Adjusted for: history of abdominal pain and diarrhea.

*- significant at p<0.05.

### *S. mansoni* infection

The overall prevalence of *S. mansoni* infection was 6.4% (CI_95%_:4.4–9.1) and the median parasite density was 48 eggs/g (IQR:24–76)(Table 3). The likelihood of having *S. mansoni* infection was high in children bathing in Mango river (aOR:2.7;CI_95%_:1.2–6.0;p = 0.02) and in girls (aOR: 2.3 CI_95%_ 1.0–5.0; P = 0.04). Other factors such as history of abdominal pain, diarrhea, bloody feces before enrolment did not demonstrate any association with *S. mansoni* infection (Table 4).

### STH infections

The prevalence of STH infections was estimated to be 32.8% (CI_95%_:28.6–37.4). Only two of the three majorSTH were found: *Trichuris trichiura* (26.3%) and *Ascaris lumbricoides* (20.1%). Hookworms were not found. However one case of a non-STH, *Enterobius vermicularis* was diagnosed. Girls (aOR:0.62;CI_95%_:0.41–0.92;p = 0.02) and younger children (aOR:0.52;CI_95%_ 0.29_–_0.97;p = 0.04) had a significant lower risk for STH infection (Table 4). Factors such as history of abdominal pain and diarrhea were not associated to STH infection (p = 0.9) (data not shown).

### *S. haematobium* infection

No infection with *S. haematobium* were found in the children’s urine.

### Co-infections

**Figure 2:**
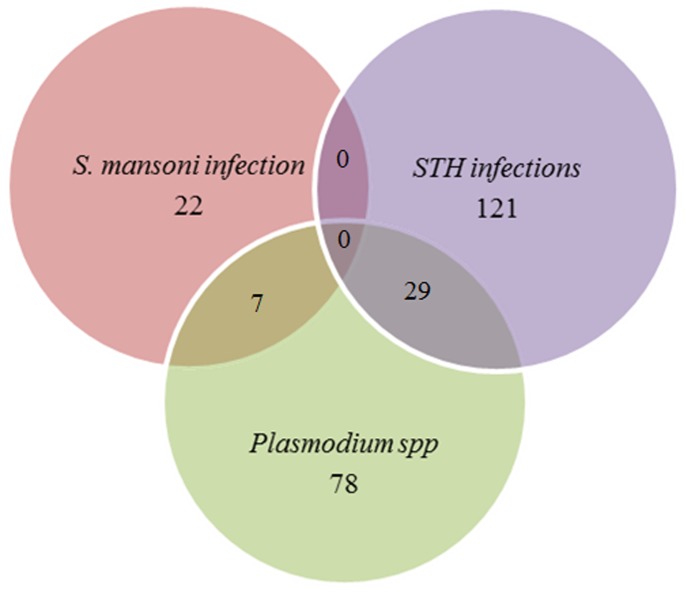
Venn diagram showing Malaria – *S. mansoni* and malaria – STH infections co-infections.

Co-infection with malaria and a helminth was common though we did not observe any *S. mansoni*-STH co-infection. The prevalence rate of malaria-*S. mansoni* and malaria-STH co-infections was respectively 1.5% (CI_95%_:0.7–3.3) and 6.4% (CI_95%_ 4.4–9.1) (fig. 2). There was no association found between helminth status and malaria infection (p = 0.3) or schistosomiasis and malaria infection (p = 0.7) (data not shown). Co-infection with at least two parasite species (Malaria-*S. mansoni*, Malaria- STH or between two STH species) was found in 79 participants (17,3%;CI_95%_:15.3–20.8).

### Nutritional status

The nutritional status of most children was within healthy parameters but a few cases of stunting (HAZ<−2) 0.16%, thinness (BAZ<−2) 0.65% and underweight (WAZ<2) 0.97% were observed (Table 1).

### Anemia

The mean hemoglobin concentration was 11.6 g/dL (SD±1.4) and was found to be lower in children with asymptomatic malaria infection compared to uninfected children (p<0.001) (table 5). Hb concentration was not lower in children with Schistosomiasis (p = 0.06) or STH infection (p = 0.12). Children with co-infections had a 1.2 g/dl lower Hb (p = 0.01) when infected with malaria-*S.mansoni*, and a 0.66 g/dl Hb lower when co-infected with STH (p = 0.02) than all not co-infected (Table 5). Other factors such as malnutrition, history of fever, history of transfusion and the intensity of parasitic infections were not correlated to the Hb concentration.

**Table 5:** Mean Hb, anaemia prevalence and predictors for anaemia in asymptomatic children attending two primary schools in Mokali heath semi- rural area, in Kinshasa, 2012.

Variables		N (%)	Total (N)	Mean Hb g/dL (SD)	P value	Anemia	cOR	P value	aOR	P value
Age	4–9	556 (90.3%)	616	11.56 (1.25)	0.2	249(44.8%)	1.0	0.1		
	10–13	60 (9.7%)		11.77 (1.16)		27(45.0%)	1		–	-
Sex	Female	298 (48.4%)	616	11.63 (1.18)	0.4	130(43.6%)	0.91	0.6	–	
	Male	318 (51.6%)		11.53 (1.29)		146(45.9%)	1			
Malaria infection	Yes	114 (18.5%)	616	10.78 (1.44)	<0.001*	81(71.0%)	3.8	<0.001*	4.5 (2.6–7.7)	<0.001*
	No	502 (81.5%)		11.76 (1.12)		195(38.8%)	1			
*S. mansoni* infection	Yes	29 (6.4%)	457	11.15 (1.12)	0.06	20(69.0%)	2.9	0.01*	2.5 (1.0–6.0)	0.01*
	No	428 (93.6%)		11.60 (1.24)		185(43.22%)	1			
*T. Trichiura* Infect.	Yes	120 (26.3%)	457	11.50 (1.22)	0.4	54 (45.0%)	1.0	0.9	–	–
	No	337 (73.7%)		11.60 (1.25)		151 (44.8%)	1			
*A. Lumbricoides* Infect.	Yes	92 (20.2%)	457	11.45 (1.13)	0.3	47 (51.1%)	1.4	0.2	–	
	No	365 (79.8%)		11.60 (1.26)		158 (43.2%)	1			
Intestinal infection status	Mixed	57 (12.5%)	457	11.26 (1.18)	0.12	34(59.6%)	1.9	0.03*	–	–
	Mono	122 (26.7%)		11.63 (1.17)		49(40.1%)	0.76	0.2	–	
	No	278 (60.8%)		11.61 (1.27)		122(43.9%)	1			
History of fever	Yes	191 (31.0%)	616	11.47 (1.26)	0.3	87 (45.5%)	1.3	1.2	–	–
	No	425 (69.0%)		11.63 (1.23)		169 (39.7%)	1			
History of transfusion	Yes	71 (11.5%)	616	11.61 (1.41)	0.8	31 (43.7%)	1.1	0.7	–	–
	No	545 (88.5%)		11.57 (1.22)		225 (41.3%)	1			
Ownership and use of Bednet	Yes and used	81 (13.1%)	589	11.50 (1.22)	0.8	36 (44.4%)	1.1	0.7	–	–
	Yes and not used	37 (6.0%)		11.65 (1.23)		14 (37.8)	0.83	0.6	–	–
	No	471 (76.5%)		11.58 (1.26)		199 (42.3%)	1			
Malaria parasite density	Very heavy	10 (8.8%)	114	10.79 (1.85)	0.7	NA				
	Heavy	40 (35.1%)		10.94 (1.27)						
	Moderate	40 (35.1%)		10.59 (1.48)						
	Low	24 (21%)		10.84 (0.83)						
*S. mansoni* infection density	Heavy	2 (6.9%)	29	11.20 (1.01)	0.3	NA				
	Moderate	3 (10.3%)		11.16 (0.80)						
	Low	24 (82.8%)		11.14 (1.21)						
Co-infection (Malaria- SCH)	Yes	7(1.5%)	457	10.37 (0.93)	0.01*	NA				
	No	450 (98.5%)		11.59 (1.23)						
Co-infection (Malaria –Helminth)	Yes	29 (6.3%)	457	10.96 (1.48)	0.02*	NA				
	No	428 (93.7%)		11.61 (1.25)						

N - Number, SD- Standard Deviation, c OR- crude Odds- Ratio, aOR- adjusted Odds-Ratio, NA- Not Applicable, SCH-Schistosomiasis.

*- significant at p<0.05.

**Figure 3:**
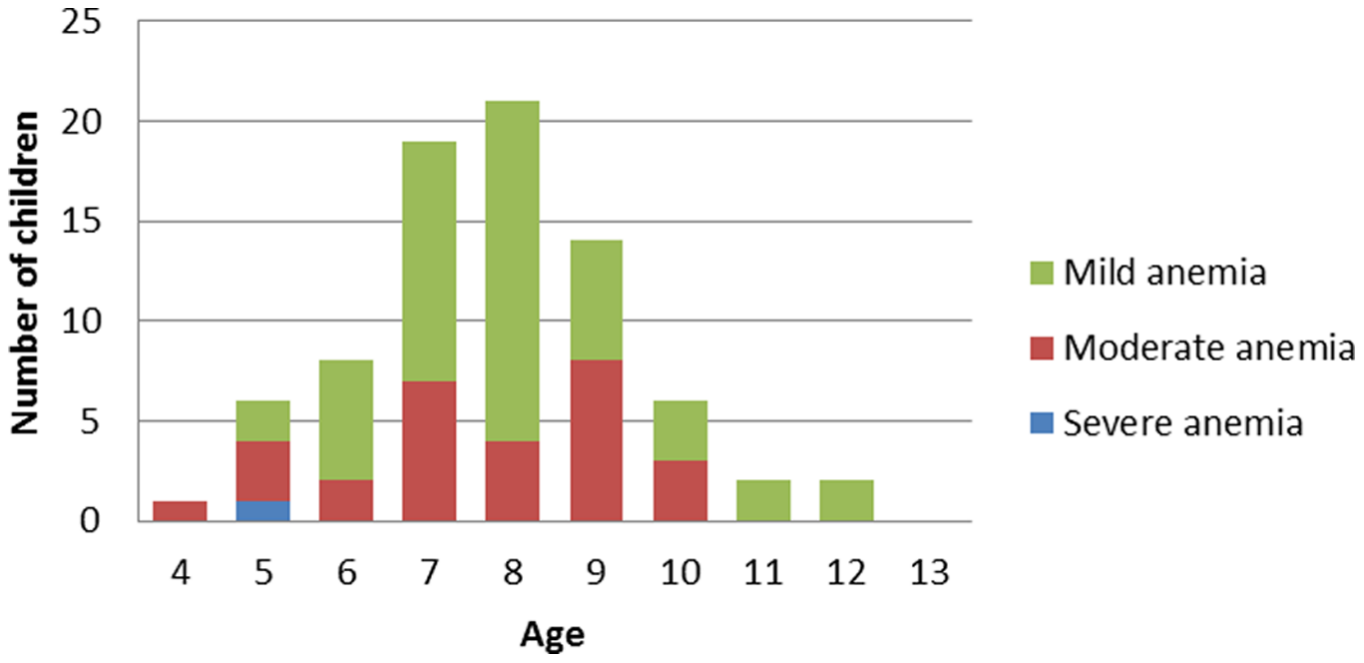
Distribution of anaemia in malaria infected children according to age in Kinshasa.

The prevalence of anemia was estimated to be 41.6% (CI_95%_:37.7–45.6) with no difference between sexes (p = 0.6). It was more common in *P.falciparum*-infected children (71.0%) than in uninfected children (38.8%) (p<0.001) (Table 4). Of these anemic schoolchildren, 193 (31.3%), 62 (10.1%), 1 (0.2%), had respectively, mild, moderate and severe anemia (table 5). In this study, mild anemia occurred more frequently in children younger than 9 years with a peak in 8 year olds (figure 3). No significant differences were found in the malaria and *S. mansoni* parasite density in the children presenting mild, moderate or severe anemia (data not shown). The multivariate regression model shows that anemia was associated with asymptomatic malaria infection (aOR:4.5;CI_95%:_2.6–7.7;p<0.001) and *S. mansoni* infection (aOR:2.5;CI_95%_: 1.0–6.0; p<0.01) independently (Table 4). No interaction between *S. mansoni* and malaria infection was observed.

## Discussion

In the Mokali Health Area, a semi-rural area of Kinshasa located in the Health Zone of Kimbanseke, the prevalence of asymptomatic malaria infection in schoolchildren was found to be 18.5%. Similar observations were made in 1981–1983 in Kinshasa, and 2000 in Kimbanseke [29]. In this study, the increased malaria risk for older children was unexpected (Table 4). The prevalence of asexual stages of *P. falciparum* in endemic areas is supposed to decrease significantly with age, because children would gradually developed some degree of immunity against the malaria parasite, as a result of repeated infections [30]. However, this observation was also reported in the Kikimi Health Zone also located in Kimbanseke zone [29]. In a study conducted in Brazzaville, a higher malaria prevalence in older children was attributed to the increased use of antimalarial drugs, particularly in early childhood [31]. There was a significant association between history of fever around the time of the enrolment and malaria parasitemia, and this agrees with a study conducted in Nigeria [32]. On the other hand, this study revealed a prevalence of symptomatic children of 3.4%, with 41.2% having a positive tick blood smear. This rate of symptomatic children at school was high and unexpected. These results suggests that malaria in school age children, thought usually asymptomatic, can result into mild and somewhat well tolerated symptoms compared to under five years children. Symptomatic children had a significantly higher malaria parasite density compared to those asymptomatic. These findings underline the complexity of the clinical presentation of *P. falciparum* infection in endemic areas.

Like malaria, STH were highly prevalent in the study population (32.8%). This could be the result of poor sanitary conditions in the Health Area of Mokali. This study recorded a prevalence of 26.2% for *T. trichiura* having the highest prevalence, followed by *A. lumbricoïdes* (20.1%). These values are significantly lower than 90% and 83.3% respectively for *A. lumbricoïdes* and *T. trichiura* reported by Vandepitte in 1960 in Kinshasa [33]. The prevalence of these two parasites declined and was found to be respectively 57% and 11% in 1980 [34]. These drastic changes in prevalence could be explained by the education and increase awareness [35]. The prevalence found in this study showed a further decrease of *A. lumbricoides* infection, however improved sanitary, access to adequate water supply and access to health care should further decrease the prevalence of STH infections.

This study also estimated the prevalence of *S. mansoni* infection to be 6.4%. This prevalence is significantly lower compared to 89.3% reported in 2012 in Kasansa Health Zone, another endemic setting for *S. mansoni* in DRC [36]. Girls were more likely to be infected than boys. This is probably because they are traditionally responsible for water related household chores in poor countries [37], therefore being more frequently in contact with contaminated water. Children who regularly bathed in the Mango river were significantly more likely to be infected than those who did not. These findings emphasize the need for extensive malacological studies in this area to identify the intermediate host species specifically in Mango river. Reported history of bloody feces, diarrhea and abdominal pain were not related to *S. mansoni* infection. Similar observation was found in Yemeni in California [38]. This could be due to the low parasite load observed in the study population (more than 80% having light parasite load). Most of the infected children were probably in the chronic phase of the disease. Therefore, they presented a low grade of acute symptoms although anemia was significantly associated with infection.

Co-infection with *P. falciparum* and *S. mansoni* occurred at very low levels (1.5%). This is consistent with findings from Kenya in 2008–09 and Ethiopia 2008–09 and Uganda 2006 [39]. However, *P. falciparum* and STH co-infections were more frequent (6.4%). No association was found between malaria infection and *S. mansoni* infection neither between malaria infection and STH infection. This is in total agreement with previously reported data from Tanzania in 2010 [40].

On the other hand, the prevalence of anemia in primary schoolchildren was found to be 41.6%. This was lower than 67% observed in Kasansa, DRC in 2012 [36]. The likelihood of having anemia was about 4 times more in malaria infected schoolchildren. Mean hemoglobin concentration was significantly lower in malaria infected children compared to uninfected children with an incremental Hb level of 0.98 g/dL. The present study as many others conducted in others settings across Africa [41], [42], demonstrated the major role played by malaria in the occurrence of anemia in schoolchildren in sub-Saharan Africa. In disagreement with other findings [43], *S. mansoni* infection was also found to be an independent risk factor for anemia in schoolchildren. No interaction was found between asymptomatic malaria infection and *S. mansoni* in regard to anaemia.

The study has a number of limitations. First, given the high rate of refusal (32.8%), which may lead to a selection bias, the reported data may not be representative of the schools surveyed. However, given that children whose parents did not consent were similar to those included in regard to age, sex and class, we have no reason to suspect that children in these two groups differed greatly in regard to other characteristics not assessed. This high proportion of refusal may indirectly suggest a negative perception of IPT or other malaria intervention in schoolchildren by the community. This underlines the urgent need to assess the perception and potential social and cultural barriers that can prevent an efficient implementation of malaria control strategies in schoolchildren. Second, asymptomatic malaria infection is mostly characterized by low grade parasitemia [44]. Conventional microscopy, the laboratory method used in the present study, is not sensitive enough to detect low-grade, asymptomatic infections. Therefore, a highly sensitive PCR-based diagnosis, which is between 2.7-fold and 8.6-fold more sensitive than conventional microscopy in detecting malaria parasites in apparently health children [45], [46], would provide a more accurate picture of asymptomatic *plasmodium spp* carriage. Finally, no hookworms were found in Mokali’s schoolchildren. This could be at least partly explained by the fact that, the Kato-Katz slides were examined after 24 hours, which situation may lead to overclearance of hookworm eggs by glycerol.

## Conclusion

This study demonstrated that *P. falciparum* infection was highly prevalent in schoolchildren of Biyela Health Zone, and along with *S. mansoni* infection, they contribute to a great extent to the occurrence of anemia. These results highlight the important role of school-based interventions, which may include: deworming, micronutrients and intermittent preventive treatment for malaria for the control of anemia among African schoolchildren.

## References

[1] World Health Organization (2001) Schistosomiasis and soil-transmitted infections. 54th World Health Assembly, agenda item 13.3 resolution WHA 54.19.

[2] Petney TN, Andrews RH (1998) Multiparasite communities in animals and humans: frequency, structure and pathogenic significance. Int J Parasitol 28: 377–93. 9559357 10.1016/s0020-7519(97)00189-6

[3] World Health Organization (2002) Prevention and control of schistosomiasis and soil-transmitted helminthiasis: first report of the joint WHO expert committees. WHO Technical Report Series. Geneva: World Health Organization. 12592987

[4] Buck AA, Anderson RI, MacRae AA (1978) Epidemiology of poly-parasitism I. Occurrence, frequency and distribution of multiple infections in rural communities in Chad, Peru, Afghanistan and Zaire. Trop Med Parasitol 29: 61–70. 644660

[5] Henning L, Schellenberg D, Smith T, Henning D, Alonso P, et al (2004) A prospective study of Plasmodium falciparum multiplicity of infection and morbidity in Tanzanian children. Trans R Soc Trop Med Hyg 98: 687–94. 15485698 10.1016/j.trstmh.2004.03.010

[6] Smith T, Felger I, Tanner M, Beck HP (1999) Premunition in Plasmodium falciparum infection: insights from the epidemiology of multiple infections. Trans R Soc Trop Med Hyg 93 Suppl 1 59–64. 10450428 10.1016/s0035-9203(99)90329-2

[7] Verhoef H (2010) Asymptomatic malaria in the etiology of iron deficiency anemia: a malariologist's viewpoint. Am J Clin Nutr 92: 1285–6. 21068352 10.3945/ajcn.110.006700

[8] White NJ, Ho M (1992). The pathophysiology of malaria. In: Advances in Parasitology. Baker, J.R., Muller, (editors). 3rd edition. New York: Academic Press 84–175.

[9] Faquin WC, Schneider TJ, Goldberg MA (1992) Effect of inflammatory cytokines on hypoxia-induced erythropoietin production. Blood 79: 1987–94. 1373333

[10] Jelkmann W, Pagel H, Wolff M, Fandrey J (1992) Monokines inhibiting erythropoietin production in human hepatoma cultures and in isolated perfused rat kidneys. Life Sci 50: 301–8. 1310133 10.1016/0024-3205(92)90338-p

[11] Hotez PJ, Kamath A (2009) Neglected tropical diseases in sub-saharan Africa: review of their prevalence, distribution, and disease burden. PLoS Negl Trop Dis 25: 3. 10.1371/journal.pntd.0000412 PMC2727001 19707588

[12] Bundy DA, Lwin S, Osika JS, McLaughlin J, Pannenborg CO (2000) What should schools do about malaria?. Parasitol Today 16: 181–2. 10782071 10.1016/s0169-4758(00)01658-6

[13] Friedman JF, Kanzaria HK, McGarvey ST (2005) Human schistosomiasis and anemia: the relationship and potential mechanisms. Trends in Parasitology 21: 386–392. 15967725 10.1016/j.pt.2005.06.006

[14] Lemaitre M, Watier L, Briand V, Garcia A, Le Hesran JY, et al (2014) Co-infection with Plasmodium falciparum and Schistosoma haematobium: Additional Evidence of the Protective Effect of Schistosomiasis on Malaria in Senegalese Children. Am J Trop Med Hyg 90: 329–34. 24323515 10.4269/ajtmh.12-0431 PMC3919243

[15] Brutus L, Watier L, Hanitrasoamampionona V, Razanatsoarilala H, Cot M (2007) Confirmation of the protective effect of Ascaris lumbricoides on Plasmodium falciparum infection: results of a randomized trial in Madagascar. Am J Trop Med Hyg 77: 1091–5. 18165528

[16] Hesran J, Akiana J, Ndiaye EI, Dia M, Senghor P, et al (2004) Severe malarial attack is associated with high prevalence of Ascaris lumbricoides infection among children in rural Senegal. Trans R Soc Trop Med Hyg 98: 397–399. 15138075 10.1016/j.trstmh.2003.10.009

[17] Shapiro AE, Tukahebwa EM, Kasten J, Clarke SE, Magnussen P, et al (2005) Epidemiology of helminths infections and their relationship to clinical malaria in southwest Uganda. Trans R Soc Trop Med Hyg 99: 18–24. 15550257 10.1016/j.trstmh.2004.02.006

[18] Midzi N, Mtapuri-Zinyowera S, Mapingure MP (2010) Consequences of polyparasitism on anaemia among primary school children in Zimbabwe. Acta Trop 115: 103–11. 20175980 10.1016/j.actatropica.2010.02.010

[19] Ezeamama EE, McGarvey ST, Acosta LP (2008) The synergistic effect of concomitant schistosomiasis, hookworm, and trichuris infections on children’s anemia burden. PLoS Negl Trop Dis 2: e245. 18523547 10.1371/journal.pntd.0000245 PMC2390851

[20] World Health Organization. World Malaria Report 2008. Who website. Available: http://www.who.int/malaria/publications/atoz/9789241563697/en/. Accessed 2014 October 14.

[21] Gething PW, Kirui VC, Alegana VA, Okiro EA, Noor AM, et al (2010) Estimating the Number of Paediatric Fevers Associated with Malaria Infection Presenting to Africa’s Public Health Sector in 2007. PLoS Medicine 7 1–12. 10.1371/journal.pmed.1000301 PMC2897768 20625548

[22] Nzuzi FL (2008) Kinshasa: Ville et Environnement, Paris, L’Harmattan.

[23] Doua JY, Matangila JR, Lutumba P, Van geertruyden JP (2013) Intermittent preventive treatment: efficacy and safety of sulfadoxine-pyrimethamine and sulfadoxine-pyrimethamine plus piperaquine regimens in schoolchildren of the Democratic Republic of Congo: a study protocol for a randomized controlled trial. Trials 24: 311. 10.1186/1745-6215-14-311 PMC4015766 24063608

[24] Katz N, Chaves A, Pellegrino J (1972) A simple device for quantitative stool thick-smear technique in Schistosomiasis mansoni. Rev Inst Med Trop Sao Paulo 14: 397–400. 4675644

[25] WHO (2002) Prevention and control of schistosomiasis and soil transmitted helminthiasis. Geneva, Switzerland: WHO Expert Committee. 12592987

[26] Okanla EO (1991) Schistosomiasis: influence of parental occupation and rural or urban dwelling on prevalence. Nig J Pure Appl Sci 6: 154–159.

[27] Worldwide prevalence of anaemia 1993–2005: WHO global database on anaemia. WHO website. Available: http://www.who.int/vmnis/anaemia/prevalence/en/. Accessed 2014 October 14.

[28] de Onis M, Habicht JP (1996) Anthropometric reference data for international use: Recommendations from a WHO Expert Committee. Am J Clin Nutr 64: 650–658. 8839517 10.1093/ajcn/64.4.650

[29] Kazadi W, Sexton J, Makengo B, Bompela WO, Matezo W (2004) Malaria in primary school children and infants in Kinshasa, Democratic Republic of the Congo: Surveys from the 1980S and 2000. Am. J. Trop. Med. Hyg (Suppl 2): 97–102. 15331825

[30] Bruce MC, Donnelly CA, Packer M, Lagog M, Gibson N, et al (2000) Age- and species-specific duration of infection in asymptomatic malaria infections in Papua New Guinea. Parasitology 121: 247–256. 11085245 10.1017/s0031182099006344

[31] Trape JF (1987b) Malaria and urbanization in Central Africa: the example of Brazzaville. Part IV: Parasitological and serological surveys in urban and surrounding rural areas. Trans R SocTrop Med Hyg 81 Suppl 2 27–33. 10.1016/0035-9203(87)90474-3 3332057

[32] Gbadegesin RA, Sodeinde O, Adeyemo AA, Ademowo OG (1997) Body temperature is a poor predictor of malaria parasitaemia in children with acute diarrhoea. Ann Trop Paediatr 1: 89–94. 10.1080/02724936.1997.11747869 9176584

[33] Vandepitte J (1988) Helminthologie médicale. Université de Kinshasa, 122 pages.

[34] Jancloes MF, Cornet P (1980) Epidemiological control of intestinal nematodoses in a rural area of Zaïre. Revue épidémiologique. Santé Publique 28: 89–103. 7465915

[35] Asaolu SO, Ofoezie IE (2003) The role of health education and sanitation in the control of helminth infection. Acta Trop 86: 283–294. 12745145 10.1016/s0001-706x(03)00060-3

[36] Kabongo M (2012) Impact of Schistosomiaisis in Kasansa Health Zone in Democratic Republic of Congo. Public Health Thesis paper 220.

[37] The World's Women (2010) Trends and Statistics. United Nations New York.

[38] Warren KS, Mahmoud AA, Cummings P, Murphy DJ, Houser HB (1974) Schistosomiasis mansoni in Yemeni in California: duration of infection, presence of disease, therapeutic management. Am J Trop Med Hyg 23: 902–9. 4451230 10.4269/ajtmh.1974.23.902

[39] Brooker SJ, Pullan RL, Gitonga CW, Ashton RA, Kolaczinski JH, et al (2012) *Plasmodium*-helminth coinfection and its sources of heterogeneity across East Africa. J Infect Dis 205 841–52. 22262792 10.1093/infdis/jir844 PMC3274378

[40] Mazigoi HD, Kidenya BR, Ambrose EE, Zinga M, Waihenya R (2010) Association of intestinal helminths and P. falciparum infections in co-infected school children in northwest Tanzania. Tanzan J Health Res 12: 299–301. 24409638 10.4314/thrb.v12i4.56152

[41] Magalhaes RJ, Clements AC (2011) Mapping the risk of anaemia in preschoolage children: the contribution of malnutrition, malaria, and helminth infections in West Africa. PLoS Med 8: 438. 10.1371/journal.pmed.1000438 PMC3110251 21687688

[42] Walker SP, Wachs TD, Gardner JM, Lozoff B, Wasserman GA (2007) Child development: risk factors for adverse outcomes in developing countries. Lancet 369: 145–157. 17223478 10.1016/S0140-6736(07)60076-2

[43] Sturrock RF, Kariuki HC, Thiongo FW, Gachare JW, Omondi BG, et al (1996) Schistosomiasis mansoni in Kenya: relationship between infection and anaemia in schoolchildren at the community level. Trans R Soc Trop Med Hyg 90: 48–54. 8730312 10.1016/s0035-9203(96)90477-0

[44] Roucher C, Rogier C, Dieye-Ba F, Sokhna C, Tall A, et al (2012) Changing malaria epidemiology and diagnostic criteria for Plasmodium falciparum clinical malaria. PLoS One 7: e46188. 23029433 10.1371/journal.pone.0046188 PMC3460864

[45] Wang B, Han SS, Cho C, Han JH, Cheng Y, et al (2014) Comparison of Microscopy, Nested-PCR, and Real-Time-PCR Assays Using High-Throughput Screening of Pooled Samples for Diagnosis of Malaria in Asymptomatic Carriers from Areas of Endemicity in Myanmar. J Clin Microbiol 6 1838–45. 10.1128/JCM.03615-13 PMC4042795 24648557

[46] Roper C, Elhassen IM, Hviid L (1996) Detection of very low level Plasmodium falciparum infections using the nested polymerase chain reaction and a reassessment of the epidemiology of unstable malaria in Sudan. Am J Trop Med Hyg 54: 325–31. 8615441 10.4269/ajtmh.1996.54.325

